# The Earliest T-Precursors in the Mouse Embryo Are Susceptible to Leukemic Transformation

**DOI:** 10.3389/fcell.2021.634151

**Published:** 2021-04-29

**Authors:** Jixin Ding, Angelo A. Cardoso, Momoko Yoshimoto, Michihiro Kobayashi

**Affiliations:** ^1^Department of Medicine, Melvin and Bren Simon Cancer Center, Indiana University School of Medicine, Indianapolis, IN, United States; ^2^Beckman Research Institute, City of Hope National Medical Center, Duarte, CA, United States; ^3^Department of Pediatrics Wells Center for Pediatric Research, Indiana University School of Medicine, Indianapolis, IN, United States; ^4^Center for Stem Cell and Regenerative Medicine, Institute of Molecular Medicine, McGovern Medical School, University of Texas Health Science Center at Houston, Houston, TX, United States

**Keywords:** notch signaling, notch intracellular domain, yolk sac, para-aortic splanchnopleura, aorta-gonad-mesonephros region, acute T cell leukemia, hematopoietic stem cell-independent hematopoiesis

## Abstract

Acute lymphoblastic leukemia (ALL) is the most common malignancy in pediatric patients. About 10–15% of pediatric ALL belong to T-cell ALL (T-ALL), which is characterized by aggressive expansion of immature T-lymphoblasts and is categorized as high-risk leukemia. Leukemia initiating cells represent a reservoir that is responsible for the initiation and propagation of leukemia. Its perinatal origin has been suggested in some childhood acute B-lymphoblastic and myeloblastic leukemias. Therefore, we hypothesized that child T-ALL initiating cells also exist during the perinatal period. In this study, T-ALL potential of the hematopoietic precursors was found in the para-aortic splanchnopleura (P-Sp) region, but not in the extraembryonic yolk sac (YS) of the mouse embryo at embryonic day 9.5. We overexpressed the Notch intracellular domain (NICD) in the P-Sp and YS cells and transplanted them into lethally irradiated mice. NICD-overexpressing P-Sp cells rapidly developed T-ALL while YS cells failed to display leukemia propagation despite successful NICD induction. These results suggest a possible role of fetal-derived T-cell precursors as leukemia-initiating cells.

## Introduction

In the hematopoietic system, Notch signaling is essential for the commitment of multipotent hematopoietic progenitors (MPP) to the T-cell lineage and it also supports cell growth, proliferation and survival at multiple stages of thymocyte development (Tanigaki and Honjo, 2007; Hozumi et al., 2008; Luis et al., 2016). *Notch 1* is essential for the emergence of hematopoietic stem/progenitor cells (HSPCs) in the mouse embryo (Kumano et al., 2003; Robert-Moreno et al., 2005) and T-cell development in the thymus (Radtke et al., 1999; Han et al., 2002). Notch provides a key regulatory signal in determining T- vs. B-lymphoid cell fate, and is involved in the progression through the early CD4^–^CD8^–^Double-negative (DN)1, DN2, and DN3 stages of thymocyte development (Schmitt et al., 2004) and in the regulation of TCR-ß rearrangement (Wolfer et al., 2002).

The constitutive activation of Notch signaling has been linked to excessive cell proliferation and arrested differentiation, contributing to the development of cancer (Aster et al., 2000). T-cell acute lymphoblastic leukemia (T-ALL) is an aggressive hematologic malignancy, comprising 15% of all newly diagnosed pediatric ALL and generally considered a high risk leukemia (Raetz and Teachey, 2016). Activating mutations of *Notch1* are observed over 70% of pediatric and 65% of adult T-ALL cases (Weng et al., 2004; Sanchez-Martin and Ferrando, 2017; Kimura et al., 2019). The Loss-of-function mutations in FBXW7 are also commonly found in T-ALL and result in inhibition of ubiquitin-mediated degradation of the activated form of Notch (Iacobucci and Mullighan, 2017; Karrman and Johansson, 2017). These mutations cause ligand independent activation and stability of the Notch intracellular domain (NICD), subsequently leading to the increased proliferation and survival of leukemic cells (Staal and Langerak, 2008). Thus, activated *Notch1* mutation plays a major pathogenetic role in human T-ALL.

ALL is the most common leukemia in children. Chromosomal translocations of leukemic cells, including *ETV6-RUNX1*, *TCF3-PBX1*, *BCR-ABL1*, and *KMT2A*, are often observed in these cases. These chromosomal aberrations are reported to have occurred *in utero* and acquire second mutations to drive leukemic transformation (Ford et al., 1993; Gill Super et al., 1994; van der Weyden et al., 2011; Hein et al., 2020). Most infant ALL belongs to B-cell leukemia, 80% of them display MLL chromosomal rearrangement and also poor prognosis (Sanjuan-Pla et al., 2015), known to arise *in utero* (Ford et al., 1993; Hein et al., 2020). The development of T-ALL in infants is extremely rare, but still exists with poor prognosis and *Notch1* mutation in infant T-ALL has also been reported (Mansur et al., 2015). Importantly, *Notch1* mutation was detected in neonatal blood spots (Guthrie test) of the child and infant T-ALL patients, suggesting the in-utero origin of infant and child T-ALL with *Notch 1* mutation, similar to infant B-ALL (Eguchi-Ishimae et al., 2008; Mansur et al., 2015).

There are many leukemia mouse models developed by overexpressing leukemic fusion proteins such as BCR-ABL. However, BCR-ABL overexpression does not always induce leukemia in any progenitor cell types; it has been reported that only pro-B cells or higher progenitors were permissive to B-ALL development (Signer et al., 2010) and that B-progenitors of fetal origin developed more aggressive leukemia with shorter latency than adult BM B-progenitors upon BCR-ABL overexpression (Montecino-Rodriguez et al., 2014). In this sense, it is well known that continuous Notch activation through over-expression of NICD leads to transformation of BM HSPCs into T-ALL, as a mouse model mimicking human T-ALL (Aster et al., 2000; Wendorff and Ferrando, 2020). Therefore, NICD-overexpression may select the leukemia initiating cells that are permissive to progress T-ALL. This notion raises the question whether fetal lymphoid precursors at pre-HSC stage can become a T-ALL initiating cell.

In the fetal hematopoiesis, it is becoming recognized that there are several waves of hematopoiesis prior to the first HSC emergence in the aorta-gonad-mesonephros (AGM) region at E10.5 (Medvinsky and Dzierzak, 1996; Montecino-Rodriguez et al., 2016; Hadland et al., 2017). Traditionally, in searching the first site of HSC emergence in the mouse embryo, lymphoid potential has been intensively investigated using organ culture and stromal cell co-culture because lymphoid potential is considered to suggest the presence of HSC potential. T- and B-lymphoid potentials have been detected in the extraembryonic yolk sac (YS) and/or para-aortic splanchnopleural (P-Sp) region at E8.25–9.5 (Godin et al., 1993, 1995; Nishikawa et al., 1998; Yokota et al., 2006; Yoshimoto et al., 2011, 2012). In addition, we have recently reported the presence of HSC-independent lymphoid progenitors in E10.5 YS and AGM region that directly repopulate only B and T cells without co-culture (Kobayashi et al., 2019). While these B-progenitors are biased to innate-immune B-1 lymphocytes, YS/P-Sp-derived T-precursors develop into CD4^+^ or CD8^+^ αβT cells in the recipient mice (Yoshimoto et al., 2012).

Here we examined leukemia propagation of the earliest T-progenitors in the YS and P-Sp by introducing active Notch signaling. We transplanted NICD-induced T cells derived from YS and P-Sp culture into lethally irradiated congenic mice and found massive T-ALL development by P-Sp-derived T cells. Those T-ALL were CD4^+^CD8^+^ double positive (DP) and highly expressed Notch-target genes. Interestingly, YS-derived cells did not develop T-ALL by NICD overexpression. These data indicate the leukemogenic potential of T-precursors at pre-HSC stage in the mouse embryo, suggesting a presence of T-ALL derived from the earliest T-precursors in the fetus.

## Methods

### Mice

C57BL/6 (B6), their congenic BoyJ, and NOD/SCID/IL2Rγc^–/–^ (NSG) mice were purchased from Jackson Laboratory and were maintained under the specific pathogen free condition. B6 mice were used for timed mating to produce E9.5 and E10.5 embryos. The embryos were harvested and their somite pair numbers were counted to confirm the proper developmental stage as previously described (Lux et al., 2008; Yoshimoto et al., 2011). The YS and P-Sp tissues were digested with 0.125% collagenase (StemCell Technologies) for 5 min. After E10, P-Sp region is called AGM region. AGM region were digested with 0.25% collagenase for 30 min at 37°C. Sublethally irradiated (150 rad) NSG neonates (day 2–3) were used for E10.5 pre-HSC transplantation. Lethally irradiated (900 rad) congenic BoyJ mice were used as recipients for transplantation of YS- and P-Sp derived NICD-induced T progenitors. Sublethally irradiated adult NSG mice were also used as recipients. The experimental procedures were approved by the Institutional Animal Care and Use Committee (IACUC) at Indiana University and the Animal Welfare Committee (AWC) at UTHealth.

### *In vitro* Cultures

YS and P-Sp cells were plated on confluent Delta-like 1-expressing OP9 stromal cells (OP9-DL1, a gift of Dr. Juan Carlos Zuniga-Pflucker, University of Toronto) (Schmitt et al., 2004) in six well plates in induction medium (αMEM, 10% FBS, and 5 × 10^–5^ M 2-mercaptoethanol) supplemented with 10 ng/ml IL-7 and 10 ng/ml Flt3 ligand. Suspended cells were collected throughout the co-culture period and the phenotype of the non-adherent cells was analyzed by flow cytometry.

### Flow Cytometry

Cells from *in vitro* culture or single cell suspension from peripheral blood (PB), spleen, BM, and thymus were stained with various surface antibodies and analyzed using LSRII (Becton Dickinson). The following antibodies were used: anti-mouse AA4.1 (AA4.1), CD19 (1D3), B220 (RA3-6B2), CD3e (145-2C11), Ter119 (TER-119), CD4 (GK1.5), CD8 (53-6.7), CD25 (PC61.5), CD44 (IM7), CCR7 (4B12), CD45.1 (A20), and CD45.2 (104). These Abs were conjugated with FITC, PE, PerCPCy5.5, PE-Cy7, APC or APC-Cy7 in various combinations.

### NICD Transduction Into YS/P-Sp Derived Cells and Transplantation

Standard retrovirus infections were performed as previously report with slight modifications (Kobayashi et al., 2017; Rodriguez et al., 2020). YS or P-Sp derived hematopoietic cells, 6–7 days after co-culture with OP9-DL1, were plated at 2.5 × 10^5^ cells per well on a 24 well plate the day before viral transduction. NICD retrovirus vector (Carlesso et al., 1999) was transduced in IMEM medium with 10% FBS, 10 ng/ml SCF, 10 ng/ml IL-7, and 10 ng/ml Flit3-ligand with the virus-containing supernatant plus Polybrene (final concentration 4 μg/mL; Sigma). A multiplicity of infection (MOI) of ≤5 was used. The cells suspended in viral supernatant were spinoculated at 1,700 rpm for 50 min, incubated at 37°C and 5% CO_2_ for an additional 8 h, then washed and plated in fresh medium overnight. A second transduction was performed on the following day using the same procedure. After a second transduction, cells were cultured on OP9-DL1 with IL7 to enhance cell expansion. One week after beginning the transduction, GFP^+^ cells were confirmed in the DN fraction as analyzed by flow cytometory and all the cells were injected into CD45.1^+^ congenic BoyJ recipient mice with 10^5^ BoyJ supportive BM cells. The transplanted mice were monitored daily and WBC count and donor-derived CD45.2^+^GFP^+^ cells were checked with recipient PB every 1–3 week beginning 4 weeks after transplantation.

### Histology

Tissues collected from non-transplanted and P-Sp-NICD T cell transplanted mice were fixed in IHC Zinc Fixative (BD Pharmingen) and embedded in paraffin. Each organ paraffin block was serially sectioned at 5 μm and stained with Hematoxylin-Eosin. The slides were examined and scored blind by our pathologist.

### Quantitative RT-PCR Analysis

Total RNA was isolated using the RNeasy kit (Qiagen, Valencia, CA, United States), and reverse transcribed into cDNA (iScript cDNA synthesis kit, Bio-Rad, Hercules, CA, United States). qRT-PCR reactions were performed using gene-specific probes either in a MX3000 system using SYBR Green chemistry (Stratagene, La Jolla, CA, United States), or in a 7900HT Fast system using Taqman probes (Applied Biosystems, Foster City, CA, United States). The sequences of gene-specific probes are following; GAPDH-F, AAGCCCATCACCATCTTCCA, GAPDH-R, TAGACTCCACGACATACTCA, Deltex-F, GCCATGTACTCC AATGGCAACAAG, Deltex-R, CGGGATGAGGTGAAAC TCCATCTT, mIL-7Rα, Mm00434295_m1 (ThermoFisher Scientific), mHes1, and Mm01342805_m1 (ThermoFisher Scientific).

### Statistical Analysis

Unpaired student-*t* test was used for statistical analysis.

## Results

### The Earliest T-Cell Precursors Were Present in the Mouse Embryo at E9.5–10.5

We previously reported that E9.5 YS and P-Sp VE-cadherin (VC)^+^ endothelial cells (ECs) have T-cell potential detected *in vitro* culture with OP9-DL1 (Yoshimoto et al., 2012). In this study, we isolated E9.5 YS and P-Sp cells and co-cultured them with OP9-DL1 stromal cells and induced CD4CD8 DP and DN T-lymphocytes. These YS- and P-Sp-derived T-cells were engrafted in the thymus and spleen of sub-lethally irradiated NOD/SCID/IL2Rγc^–/–^ (NSG) neonates as naive and memory T cells expressing various TCR repertoires. We also found donor-derived CD3^+^ T-cells in the PB when E10.5 YS was directly injected into NSG neonates (Yoshimoto et al., 2012). Thus far, there was no report to detect transplantable T cells in the YS or AGM region without co-culture, therefore, we further explored the lymphoid-repopulating ability of E10.5 YS and AGM cells (Kobayashi et al., 2019). When total YS/AGM cells or VE-cad^+^CD45^–^c-kit^+^ cells (from 2.3 to 10 embryo equivalent cells) were injected into sub-lethally irradiated NSG neonates without *in vitro* culture step, only donor-derived T cells were detected in the PB of some recipient mice (4 out of total 32 AGM-transplanted mice and three out of total 23 YS-transplanted mice (Supplementary Figures 1A,B; Kobayashi et al., 2019). Those recipients’ spleen showed predominant T-cell repopulation (CD4^+^ and/or CD8^+^ cells) and a few B cells (Supplementary Figures 1B,C). Importantly, these B-cells were B-1 and marginal zone B-cells, but not B-2 cells (Supplementary Figure 1C; Kobayashi et al., 2019). In the recipient BM, whereas AGM- and YS-derived CD4^+^ or CD8^+^ T-cells were detected, donor-derived Mac1^+^ cells were barely detected (Supplementary Figures 1B,D), indicating E10.5 YS and AGM pre-HSC population contains T-cell biased repopulating cells. These results also suggest that E9.5 VC^+^ hemogenic ECs produce T-lymphoid precursors at E10.5.

### DN3 Cells Derived From E9.5 P-Sp and YS Express CCR9 and Showed Efficient Thymus Engraftment

**FIGURE 1:**
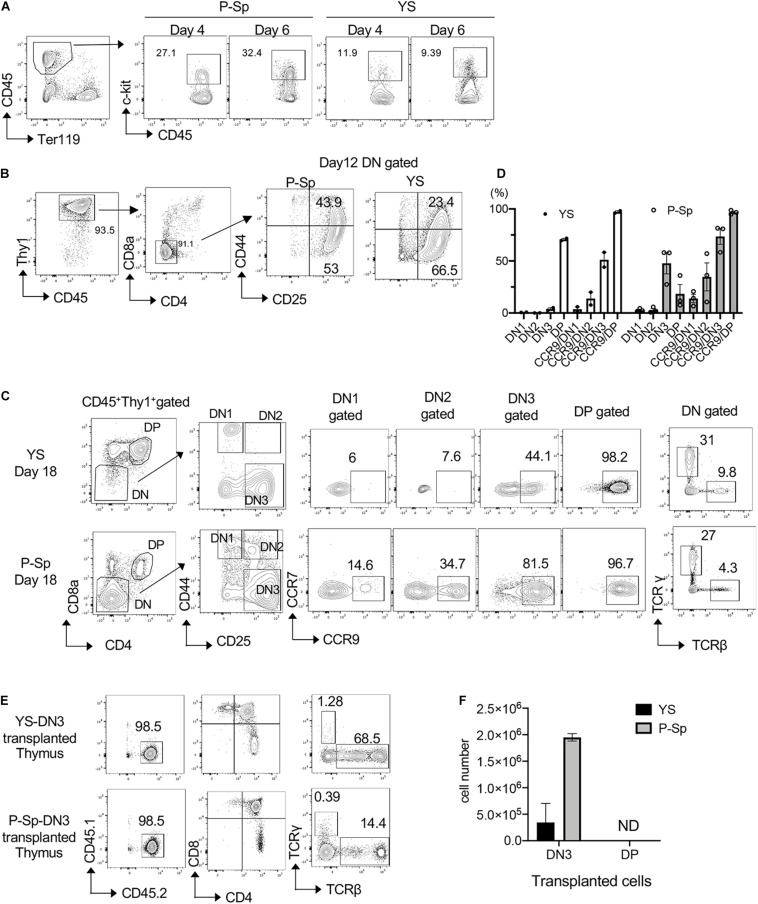
Induction of DN2 phenotype by NICD transduction. **(A)** E9.5 YS and P-Sp were cultured on DL1-OP9, and CD45^+^c-kit^+^ hematopoietic progenitors were measured on different time points, one of the three independent experiments shown. **(B)** Representative FACS plots showing DN phenotype on day 12 of YS and P-Sp co-culture on OP9-DL1. **(C)** CCR9 expression in each DN populations of YS and P-Sp cells co-cultured with DL1-OP9 for 18 days. One of the three independent experiments is depicted. TCRβ and TCRγ expressions in DN cells were also confirmed. **(D)** The percentage of DN stages and CCR9^+^ cells in each DN population of YS and P-Sp co-culture with OP9DL1, depicted in panel **(C)**. Thymus engraftment of DN3 cells from YS- and P-Sp co-culture with OP9-DL1 cells **(E)** and their repopulated cell numbers in the recipient thymus **(F)**.

Because T-lymphoid potential using OP9-DL1 culture is found in the YS and P-Sp at E9.5, 2 days before HSC detection in the AGM region, we asked their leukemic potential by overexpressing NICD (Carlesso et al., 1999). First, we observed time course of T-cell development from E9.5 YS and P-Sp cells in the OP9-DL1 culture by flow cytometric analysis to determine the timing of NICD induction. We found CD45^+^c-kit^+^ hematopoietic progenitors around day 4–7 of co-culture (Figure 1A). On day 12, CD45^+^Thy1^+^DN population was still dominant and CD25^+^CD44^+^ DN2 and CD25^+^CD44^–^DN3 cells were detected (Figure 1B). We confirmed the expression of CCR9, a homing receptor for seeding the thymus, in each DN population, in addition to TCRβ and TCRγ expression (Figure 1C). We found that CCR9 expression was the most evident in DN3 and DP populations (Figures 1C,D). Therefore, we first tested if CCR9^+^ cells engraft in the recipient thymus. We sorted DN3 and DP cells from YS and AGM culture and injected 1 × 10^6^ DN3 or DP cells into sublethally irradiated NSG neonates (Figures 1E,F). Two weeks after transplantation, we confirmed that only DN3 cells were engrafted in the recipient thymus, and found the thymus repopulated by P-Sp-DN3 cells was bigger than that repopulated by YS- DN3 cells (Figure 1F). Thus, we checked the DN3 CCR9 expression as an indicator of transplantable donor cell type during the co-culture.

### E9.5 P-Sp Derived T Lymphocytes Developed Leukemia Upon Continuous Notch Activation

**FIGURE 2:**
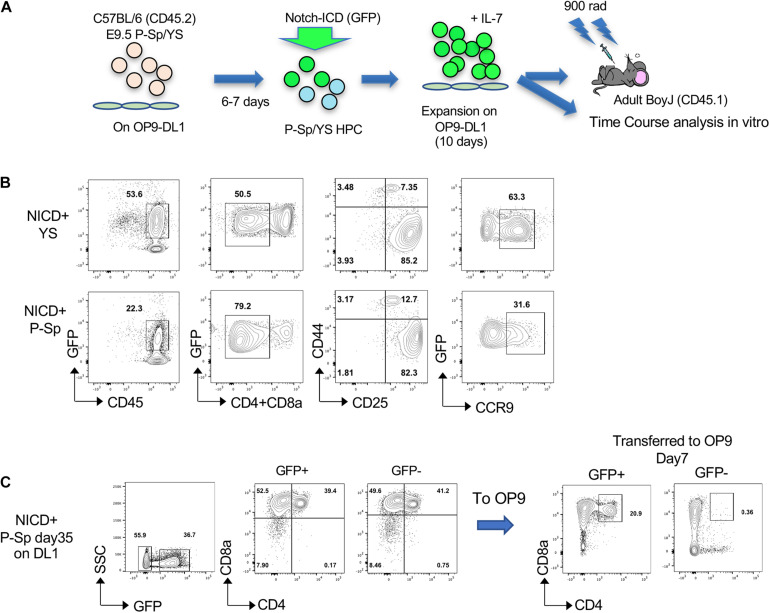
NICD introduction into hematopoietic progenitors derived from YS and P-Sp in the OP9-DL1 culture. **(A)** Schematic experimental stream for NICD induction and Transplantation. **(B)** NICD-induced GFP^+^ DN cells express CD25 and CCR9 on day 12 of co-culture with OP9-DL1 before transplantation. **(C)** P-Sp cells with NICD were harvested at day 35 and GFP^+^ or GFP^–^ cells were sorted and transferred onto OP9 (without DL1), subsequently analyzed after day 7 (right panel).

**FIGURE 3:**
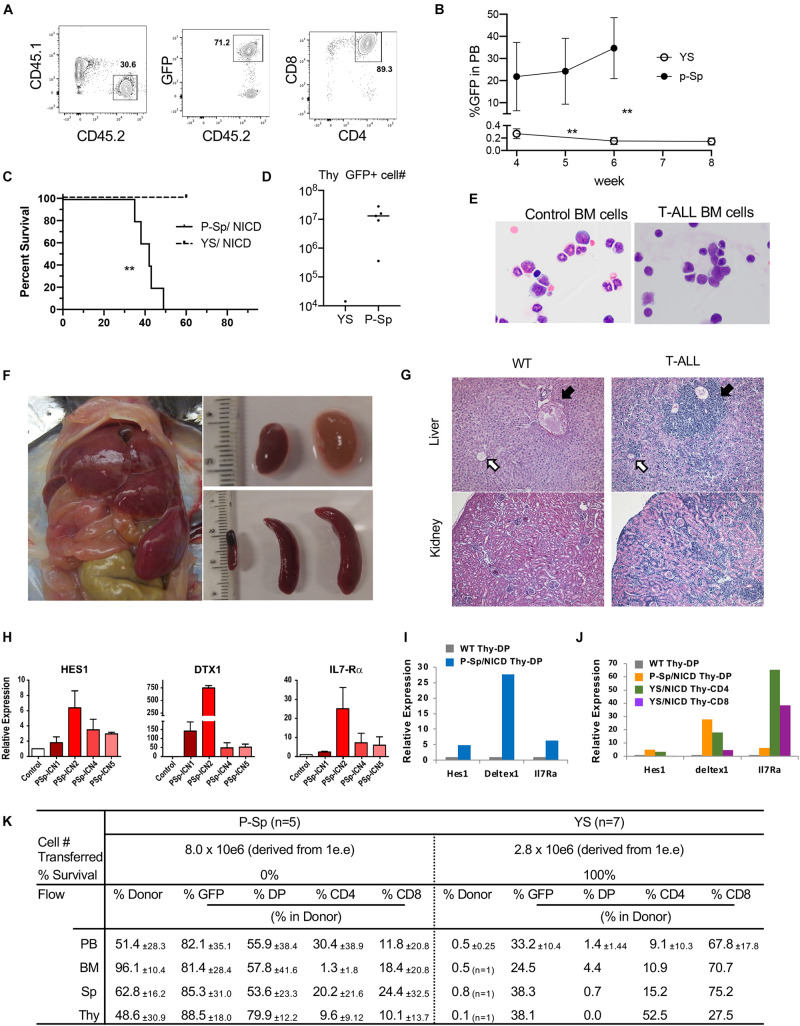
Leukemia propagation by the cultured P-Sp-NICD, not YS-NICD. **(A)** Representative dot plots of recipient PB repopulated with P-Sp-NICD at 6 weeks after transplantation. **(B)** Percentage of GFP^+^CD45.2 cells in recipient PB are plotted (*n* = 5–7, **P < 0.01). **(C)** Survival curve are depicted (**P < 0.01). **(D)** Thymus engraftment by P-Sp-derived NICD-GFP+ T-ALL cells. **(E)** May–Giemsa staining of ctr BM cells and recipient BM cells with P-Sp-NICD are shown. **(F)** Liver, spleen, and kidney are markedly enlarged. **(G)** Upper panel: liver, leukemic cells infiltrate in the portal area and around central vein as well as in the liver sinusoids. Black arrow: portal area, white arrow: central vein. Lower panel: kidney, leukemic cell infiltration was observed. **(H–J)** Upregulations of Notch targets in leukemic cells were measured by qPCR. Each RNA from recipient spleen were applied and compared to normal BM cells **(H)**. Those targets in leukemic thymic DP cells were compared to normal thymus DP cells **(I)**. Small populations of GFP^+^NICD-YS-derived CD4^+^ or CD8^+^ T-cells were sorted and evaluated for Notch targets **(J)**. **(K)** P-Sp- and YS-derived cells engrafted in the recipient organs including PB, BM, spleen, and thymus.

In order to determine if YS/P-Sp-derived T-precursors possess transforming capability similar to adult BM HSPC, NICD was retrovirally introduced into YS and P-Sp cells 6–7 days after co-culture with OP9-DL1, at the timing when CD45^+^c-kit^+^ HPCs were produced in the culture (Figures 1A, 2A). After NICD induction, NICD-GFP expressing YS- and P-Sp-derived hematopoietic progenitor cells were expanded on OP9-DL1 again up to 6 days and were injected into lethally irradiated BoyJ recipient mice together with BoyJ BM supporting cells (Figure 2A). We confirmed that the injected cells were mostly DN3 cells, containing phenotypic leukemia initiating cells (Figure 2B) (Tremblay et al., 2010; Gerby et al., 2014). We also confirmed the CCR9 expression in GFP^+^ cells (Figure 2B). As soon as 5 weeks after transplantation of NICD-expressing YS- and P-Sp-derived cells into recipient BoyJ mice, recipients transplanted with P-Sp-derived cells started to show leukemic symptoms such as high WBC, comprised largely of CD4^+^CD8^+^ GFP^+^ cells in the PB (Figures 3A,B). The mice subsequently progressed to a moribund appearance within a few days. In contrast, NICD-YS-derived T-cells failed to give rise to leukemic cells (Figures 3B,C,K). Thus, transplantation of NICD-P-Sp cells led to a marked decrease in survival of transplanted recipients due to T-ALL progression compared to NICD-YS cell transplanted hosts (Figure 3C). Mice receiving NICD-P-Sp cells became moribund with leukemia with a mean latency of 40 days, while mice repopulated with NICD-YS cells did not develop any signs of disease during the 18 weeks observation. All the mice transplanted with NICD P-Sp-derived cells showed a large thymus with donor-derived GFP^+^DP cells whereas NICD YS-derived cells failed to repopulate recipient thymus (Figures 3D,K). More than 95% of the leukemic BM cells comprised of donor-derived DP GFP^+^ cells (Figure 3E,K). The recipients’ liver and kidney were enlarged and also extensively infiltrated with leukemic cells (Figures 3F,G). Quantitative PCR analysis of Notch targets from total BM cells of leukemia mice revealed upregulations of Hes1, Deltex1 (Targets of Notch signaling) and IL7Rα compared to normal BM cells (Figure 3H), in line with the reports that upregulation of IL7Ra is often observed in human T-ALL cells from patients (Zenatti et al., 2011). We also compared thymus DP cells, a normal counterpart of the leukemic T-cells, to P-Sp derived leukemic cells in the recipient BM cells (Figure 3I). Notch target gene expression was much higher in the leukemic cells than in the normal DP thymic cells, showing that continuous exogenous NICD signals induced leukemia in P-Sp-derived T-cells. When we examined the recipients transplanted with NICD-YS derived T-cells, we found GFP^+^ CD4^+^, CD8^+^, and DP cells in the recipient BM but they did not proliferate (Figure 3K). Those NICD-YS derived CD4^+^ and/or CD8^+^ cells showed an upregulation of IL7R at a higher level than NICD-P-Sp derived T-cells (Figure 3J). Thus, although the YS contain autonomously developing T-cell precursors that can engraft in recipient mice, they were not permissive for NICD-induced leukemia. This is in contrast to P-Sp derived cells that contributed in every animal to leukemic development upon continuous NICD expression.

In addition, we continued NICD-P-Sp cell culture on OP9-DL1 up to 35 days (Figure 2C) because P-Sp-derived cells showed extensive proliferation *in vitro*. To test if this extensive proliferation ability is cell intrinsic due to NICD-overexpression, we removed external Notch signaling by transferring NICD-overexpressing cells onto regular OP9 cells that do not express DL1 (Figure 2C). After 7 days, NICD-GFP^+^ population kept DP cells while GFP^–^ population lost DP cells and shifted to CD8SP. These *in vitro* results indicate the cell-autonomous proliferation of P-Sp-derived DP cells by NICD-overexpression.

### E10.5 AGM Cells Possess Leukemic Potential Upon NICD Overexpression

**FIGURE 4:**
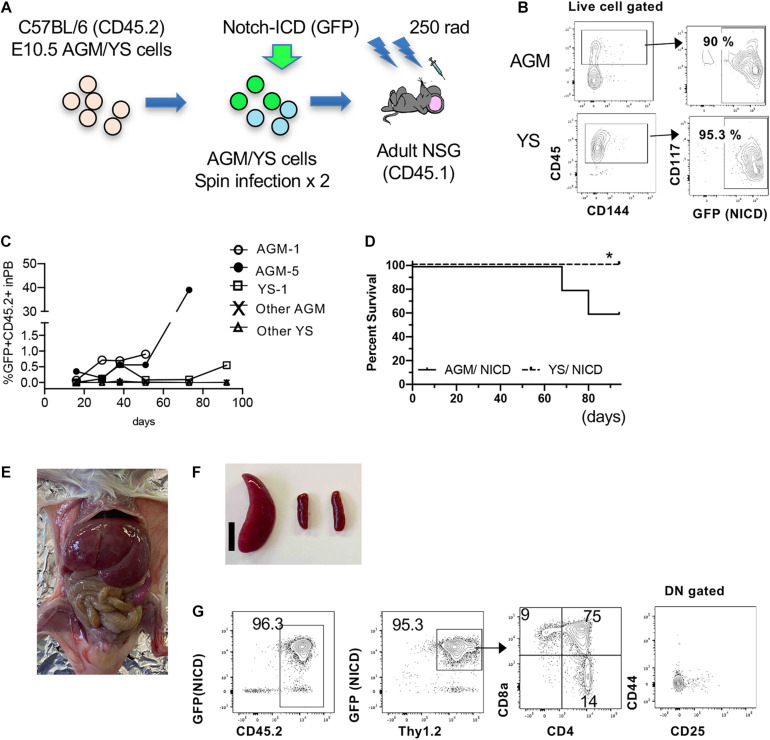
Leukemia propagation by the freshly isolated AGM-NICD. **(A)** Schematic experimental stream for NICD induction into freshly isolated AGM/YS cells and Transplantation (without co-culture). **(B)** CD45^+^AGM and YS cells showed more than 90% NICD-GFP induction. **(C)** GFP+CD45+ donor cell percentage in the recipient PB. **(D)** Survival curve of mice transplanted with AGM-NICD or YS-NICD cells is depicted (*N* = 5 each). * YS-derived cells showed GFP^+^CD4 proliferation in one recipient spleen. **(E,F)** Leukemic mice transplanted with AGM-NICD cells showed enlarged liver and spleen. Bar: 1 cm. **(G)** FACS plots of the leukemic spleen derived from AGM-NICD cells.

Because E10.5 AGM cells contain T-cell repopulating cells (Supplementary Figure 1) (Kobayashi et al., 2019), we next asked if freshly isolated AGM cells can transform T-ALL by overexpressing NICD (Figure 4A). We dissected and digested E10.5 YS and AGM cells and retrovirally transduced NCID. The following day, we transplanted NICD-expressing YS and AGM cells into sublethally irradiated adult NSG mice (1 e.e/recipient mouse, *N* = 5 for each NICD-expressing YS and AGM cells). Before transplantation, we confirmed that NICD-GFP was successfully transduced into YS and AGM cells (Figure 4B). We examined GFP^+^ donor cells in the PB of the recipient mice after transplantation over time. Most recipient mice showed less than 1% of donor cells. Only one mouse transplanted with NICD-expressing AGM cells showed increased GFP^+^CD45.2^+^ cells (Figure 1C). Interestingly, only AGM cells expressing NICD developed T-ALL in two out of five transplanted mice with longer latency compared to cultured E9.5 NICD-expressing P-Sp cells, while YS cells did not develop leukemia without showing any morbidity within the observation period (Figure 4D). The NICD-AGM-derived leukemic mice showed enlarged liver and spleen (Figures 4E,F). The leukemic spleen was occupied with CD4^+^CD8^+^GFP^+^ cells (Figure 4G), but thymus was not detected in any recipient mice. Recipient mice that did not develop T-ALL showed no donor-derived cells in the PB. Of note, one recipient mouse transplanted with NICD-YS cells was found to have an enlarged spleen when it was terminated (Supplementary Figure 2A). These cells were GFP^+^CD4^+^ cells, but not CD4^+^CD8^+^, seemed to have developed unusual transformation of CD4^+^ cells. While AGM-derived T-ALL cells occupied the recipient BM, YS-derived GFP^+^CD4^+^ or GFP^+^CD8^+^ cells were detected at a small percentage of the recipient BM (Supplementary Figure 2B). Taken together, E10.5 AGM cells possess T-ALL transforming ability.

## Discussion

In this study, we demonstrated that E9.5 P-Sp-derived cells co-cultured with OP9-DL1 possess T-ALL-initiating potential by overexpressing NICD. Traditionally, T-lymphoid potential has been detected in the YS and/or P-Sp region as early as at a pre-circulation stage (E8.25) using *ex vivo* thymic organ culture or OP9-DL1 co-culture (Nishikawa et al., 1998; Cumano et al., 2001; Yoshimoto et al., 2012). These reports have shown the T cell potential of the tested cells *in vitro* culture but never displayed the presence of committed T-progenitors. However, we have recently shown the presence of T-precursors among VC^+^c-kit^+^ HSC-precursors (pre-HSCs) in E10.5 YS and AGM region, which engrafted in the immunodeficient mice without co-culture (Kobayashi et al., 2019). Therefore, it is assumed that hemogenic ECs that have T-cell “potential” at E9.5 or before give rise to transplantable T-precursors by E10.5. Thus, we propose the possibility that fetal T-precursors at a pre-HSC stage can develop leukemia in postnatal life. However, we have not determined which cell types were responsible for the T-ALL propagation *in vivo*. Because E9.5 P-Sp cells reportedly contain “pro-HSCs” that can gain a transplantable ability by aggregation culture with OP9 (Rybtsov et al., 2014), it is possible that T-ALL initiating cells at E9.5 P-Sp developed *via* HSCs in the OP9-DL1 co-culture. However, considering the fact that mono T-cell repopulating ability was detected in AGM region in mice and humans (Ivanovs et al., 2011; Kobayashi et al., 2019) and that freshly isolated E10.5 AGM cells developed leukemia upon NICD overexpression, it is also plausible that HSC-independent T-cell precursors may have developed leukemia. A recent report examining gene signatures of human embryonic thymus, AGM, fetal liver, and fetal blood using single-cell RNA-sequencing has identified the pre-thymic lymphoid progenitors in the AGM region (Zeng et al., 2019). These data suggest that AGM region contains T-precursors that seed the fetal thymus in humans, and we propose that the T-ALL initiating cells found in the P-Sp region may belong to the initial wave of thymus seeding cells. Further investigation is required to determine the leukemic initiating cells in the embryo.

In the OP9-DL1 culture, P-Sp cells showed greater proliferation than YS cells. In general, the erythro-myeloid capacity is more abundant in the YS while more lymphoid and HSC potential is detected in the P-Sp. (Nishikawa et al., 1998; Cumano et al., 2001; Yokota et al., 2006; Yoshimoto et al., 2012). The difference of hematopoietic capacity might have contributed to the different outcome of leukemia development in this study. Another fact to be considered may be the target cell population of NICD transfection. It is noted that c-kit^+^CD45^+^ percentage was less in the YS than P-Sp in the co-culture (Figure 1A), which might reflect the progenitor numbers permissive to leukemic transformation.

We confirmed YS- and P-Sp-derived DN3 cells engrafted in the recipient thymus, and transplanted NICD-expressing cells included CCR9^+^DN3 cells, in line with the previous report that DN3 contains self-renewing leukemic cells in a T-ALL mouse model (Tremblay et al., 2010; Gerby et al., 2014). In addition, NICD-expressing P-Sp T-ALL cells in the leukemic mice showed high IL7R expression, which is in line of human T-ALL. Human T-ALL cells collected from patients at diagnosis often express IL7Ra (Zenatti et al., 2011). In addition, gain of function mutations in *IL7R* have been found in 10% of T-ALL cells, which induce Jak1/Jak3 and STAT5 activation (Ribeiro et al., 2018). It has also been reported that IL7R is essential for T-ALL development (González-García et al., 2019). Thus, high IL7R expression, known to be activated by *Notch 1* (Weng et al., 2004), is critically important for human T-ALL development. Accordingly, high IL7R-expressing T-ALL cells developed from P-Sp region by *Notch1* overexpression seem recapitulate human T-ALL. However, NICD YS-derived T-cells failed to progress leukemia despite successful NICD induction and higher IL7R expression. It is generally considered that leukemogenic events are not sufficient to induce leukemia in all blood cells; rather, they need to occur in a selective hematopoietic lineage and at a specific progenitor stage in order to develop leukemia (Signer et al., 2010). Therefore, the difference between YS- and P-Sp-derived T-cells may be a key to understanding the leukemogenic capacity of embryonic T-cells that induce pediatric leukemias.

Recent advance in the analysis of hematopoietic development in the mouse embryo has established a new paradigm of several waves of fetal hematopoiesis that initiate before HSC emergence, which may last longer than previously considered (Montecino-Rodriguez et al., 2016; Palis, 2016; Dzierzak and Bigas, 2018). For example, tissue-resident macrophages (e.g., brain microglia) and a part of mast cells are derived from the early YS progenitors and function even in the adult (Gomez Perdiguero et al., 2015; Gentek et al., 2018). It has also been indicated that several waves of thymopoietic cells are present during fetal to neonatal periods (Ikuta et al., 1990; Ramond et al., 2014; Montecino-Rodriguez et al., 2018), some of which are originated from early embryonic stages, presumably of HSC-independent. Montecino-Rodriguez et al. (2018) have shown that, using PU.1 hypomorphic embryos, the initial wave of thymopoietic cells is less dependent on PU.1 compared to the adult T-cell development and showed different gene expression patterns between fetal and adult T-cell progenitors. Ramond et al. (2014) also reported at least two waves of thymus seeding progenitors and their different properties in terms of Vγ3 generation and the cell cycle. Furthermore, Zeng et al. (2019) has reported the single-cell RNA-sequencing of various hematopoietic organs at different developmental stages in human embryo and has identified a distinct type of pre-thymic lymphoid progenitors in the AGM region. Although it remains unknown whether these embryonic early T-progenitors are derived from HSCs, their analysis segregating several ETP subsets suggests the presence of several waves of T-progenitor emergence even in the human embryo. In this sense, it has been reported that infant T-ALL has distinct genetic and epigenetic features compared to childhood T-ALL (Doerrenberg et al., 2017). Since *Notch1* mutation has been detected in neonatal blood spots in both infant and child T-ALL cases, these two pediatric T-ALL seems to be derived from prenatal period but could be from different waves of fetal hematopoiesis.

In conclusion, we found a leukemia-initiating capacity in the earliest T-precursors in P-Sp/AGM region prior to HSC emergence. It is possible that YS- and P-Sp/AGM produce different T-cell waves with different biological signatures. Further investigation is required to determine whether these fetal T-cell waves contribute to T-ALL propagation.

## Data Availability Statement

The original contributions presented in the study are included in the article/Supplementary Material, further inquiries can be directed to the corresponding author/s.

## Ethics Statement

The animal study was reviewed and approved by Institutional Animal Care and Use Committee at Indiana University and UTHealth at Houston.

## Author Contributions

JD performed the experiments and wrote the manuscript. AC provided the reagents, interpreted, and discussed the results obtained. MY and MK conceived and performed the experiments and wrote and edited the manuscript. All authors contributed to the article and approved the submitted version.

## Conflict of Interest

The authors declare that the research was conducted in the absence of any commercial or financial relationships that could be construed as a potential conflict of interest.
