# Supplementary material for: The Earliest T-Precursors in the Mouse Embryo Are Susceptible to Leukemic Transformation
Source: Front Cell Dev Biol. 2021 Apr 29;9:634151. doi: 10.3389/fcell.2021.634151 (PMC8117020; doi:10.3389/fcell.2021.634151)
Supplement: Supplementary file 1 [file Data_Sheet_1.PDF]

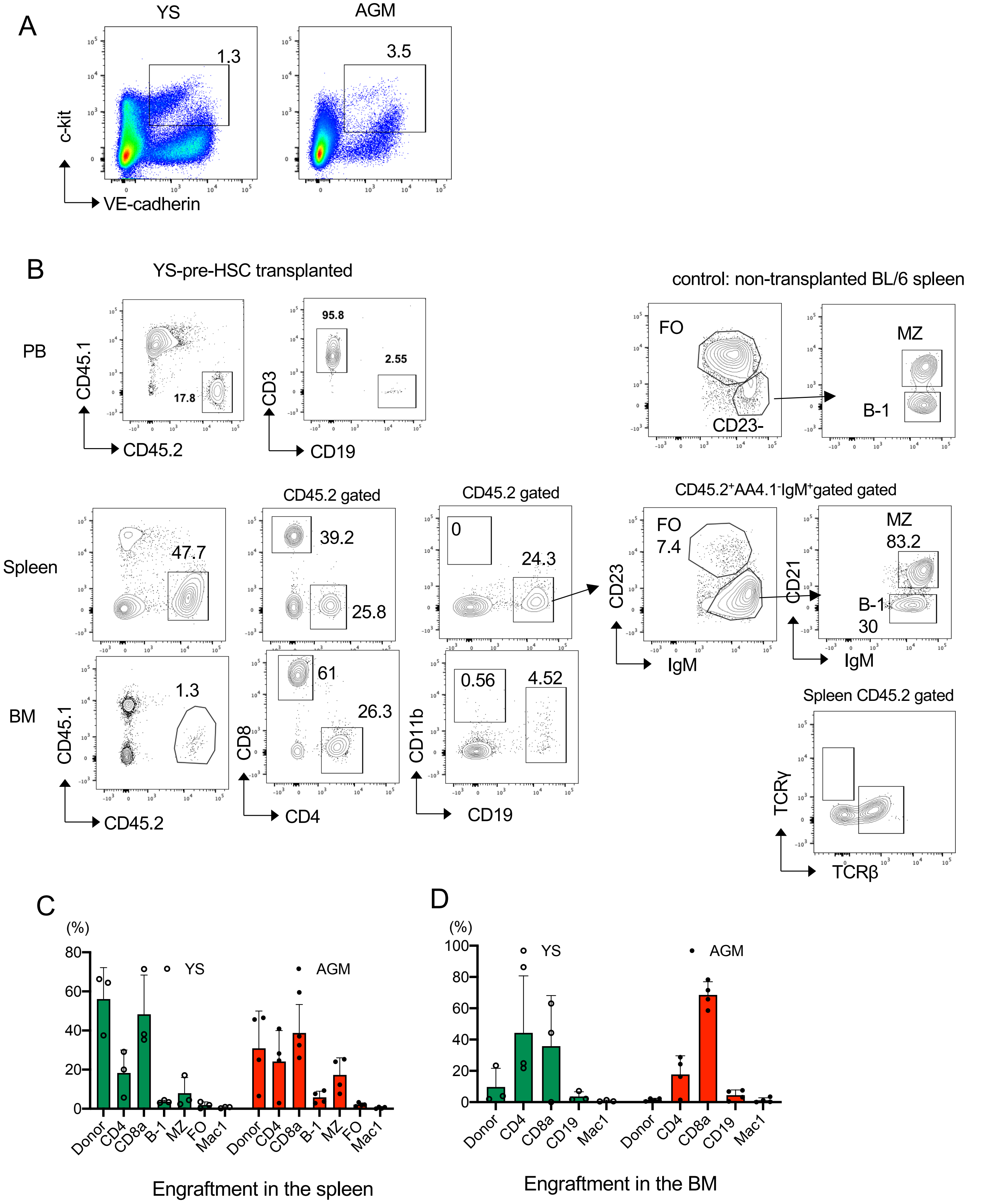

A
YS
AGM
1.3
3.5
c-kit
VE-cadherin
B
YS-pre-HSC transplanted
control: non-transplanted BL/6 spleen
PB
CD45.1
CD45.2
17.8
95.8
2.55
CD3
CD19
FO
CD23-
MZ
B-1
Spleen
BM
CD45.2 gated
CD45.2 gated
CD45.2+AA4.1-IgM+gated gated
47.7
39.2
25.8
0
24.3
FO
7.4
MZ
83.2
B-1
30
CD23
IgM
CD21
IgM
1.3
61
26.3
0.56
4.52
CD8
CD4
CD11b
CD19
Spleen CD45.2 gated
TCRγ
TCRβ
C
(%)
YS
AGM
Donor
CD4
CD8a
B-1
MZ
FO
Mac1
Engraftment in the spleen
D
(%)
YS
AGM
Donor
CD4
CD8a
CD19
Mac1
Engraftment in the BM

Figure S1

Figure S1. E10.5 YS and AGM HSC-precursor populations showed predominant T-cell repopulation ability in the recipient BM.

(A) Representative FACS plots of VC$^+$c-kit$^+$ cells from E10.5 YS amd AGM. (B) The PB (upper panel), spleen (middle panel), and BM (lower panel) of the recipient mice transplanted with VC$^+$ cells from E10.5 YS, showing CD4$^+$ or CD8$^+$, TCRβ$^+$ T cell dominant repopulation (YS, n=3, AGM, n=4). (C, D) The percentage of donor-derived T- and B- cells detected in the recipient spleen (D) and BM (D) that shows T cell dominant repopulation 4-6 months after transplantation.

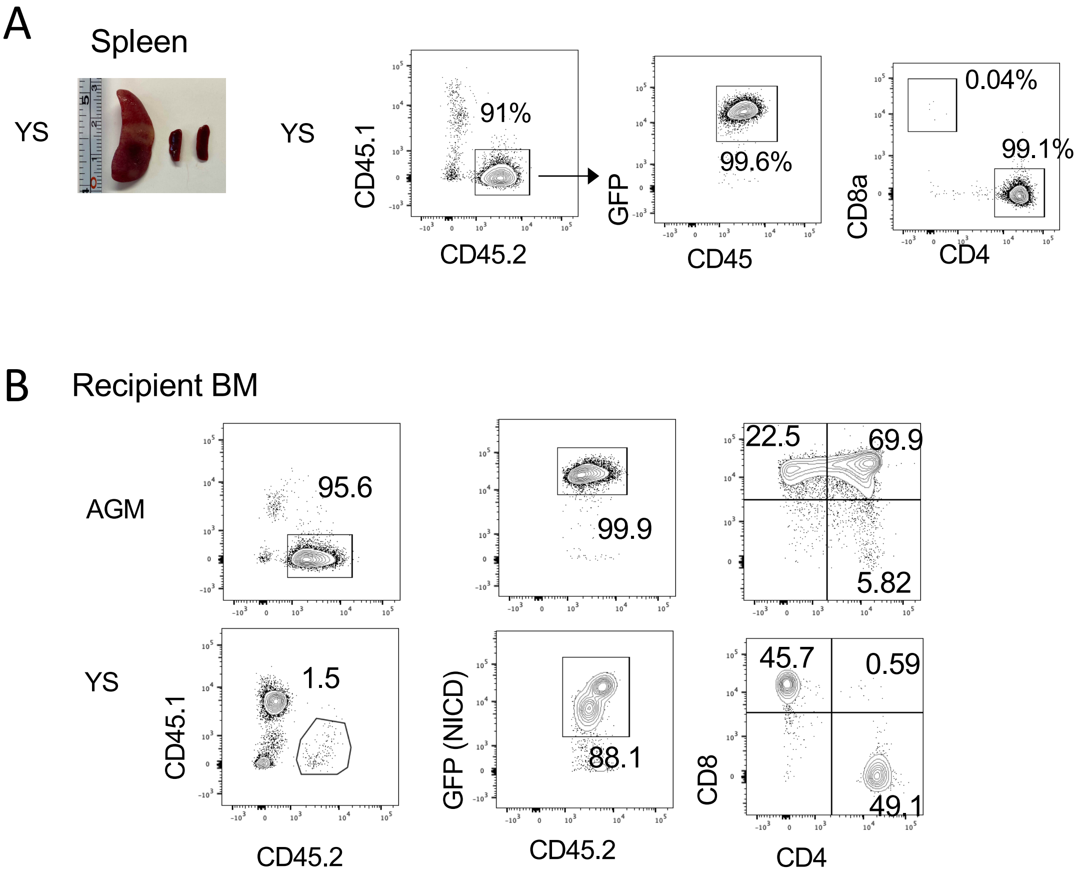

Figure S2. E10.5 YS-derived T cells did not proliferate in the recipient BM (related to Fig. 4). While NICD-expressing AGM cells developed T-ALL in the recipient mice, NICD-expressing YS cells proliferated only in the recipient spleen (A) but not in the BM (B) in only one out of 5 recipient mice.

Figure S2
